# Predictive value of combined electrocardiographic and echocardiographic parameters for major adverse cardiovascular events in patients with established coronary artery disease: a single-center retrospective cohort study

**DOI:** 10.3389/fmed.2026.1881733

**Published:** 2026-07-29

**Authors:** Qin Wu, Gang Chen, Jian Chang

**Affiliations:** 1 Department of Electrocardiography, Jingxian Hospital, Xuancheng, China; 2 Cardiology Department, Jingxian Hospital, Xuancheng, China; 3 Ultrasound Department, Jingxian Hospital, Xuancheng, China

**Keywords:** cohort study, coronary artery disease, ECG, echocardiography, major adverse cardiovascular events, risk prediction

## Abstract

**Objective:**

To explore the predictive value of combining conventional ECG parameters (P-wave dispersion, Sokolow-Lyon voltage) and echocardiographic parameters (left atrial volume index, LAVI) for major adverse cardiovascular events (MACE) in coronary artery disease (CAD) patients.

**Methods:**

From January 2024 to February 2026, 306 angiographically confirmed CAD patients were enrolled. All underwent standard 12-lead ECG and transthoracic echocardiography at admission. Over a median follow-up of 15.2 months, 50 patients developed MACE and 256 remained event-free. Baseline ECG (P-wave dispersion, QTc, Sokolow-Lyon voltage) and echocardiographic parameters (LAVI, left ventricular ejection fraction, left ventricular mass index) were compared. Independent predictors were identified by multivariate Cox regression, and a combined model was built. Incremental value was assessed by C-index, net reclassification improvement (NRI), and integrated discrimination improvement (IDI). The study was approved by the institutional review board.

**Results:**

The MACE group had higher P-wave dispersion, LAVI, and left ventricular mass index, and lower Sokolow-Lyon voltage and LVEF (all *p* < 0.05). After multivariate adjustment, increased P-wave dispersion and greater LAVI were independent predictors of MACE (both *p* < 0.05). The combination model (P-wave dispersion + LAVI) significantly outperformed the basic clinical model (age, sex, diabetes, prior MI, eGFR) in risk discrimination (C-index, NRI, IDI all *p* < 0.05). Sensitivity analyses confirmed robustness.

**Conclusion:**

Routinely available ECG-derived P-wave dispersion and echocardiographic LAVI are independent, complementary predictors of MACE in CAD patients. Integrating these two parameters into a simple risk model significantly enhances risk discrimination and reclassification, providing a practical, cost-effective tool for individualized management.

## 1 Introduction

Coronary artery disease (CAD) is one of the leading causes of morbidity and mortality worldwide, and its clinical management continues to evolve. However, accurately identifying patients with significantly increased risk of long-term adverse events remains a core challenge in clinical practice. Conventional stress tests, such as dobutamine stress echocardiography, have shown inconsistent results with positive changes in electrocardiogram (ECG) but no clear segmental wall motion abnormalities (WMAs) on echocardiography, which have been considered false positives by some scholars (1, 2); However, increasing evidence suggests that these “ECG positive/image negative” abnormal reactions are not entirely clinically significant and are clearly associated with an increased risk of major adverse cardiovascular events (MACE) in female patients, which cannot be ignored (3). In the broader CAD spectrum, the value of ECG parameters goes far beyond ischemia diagnosis. For example, the abnormal indicator of atrial electrical activity, P-wave dispersion (PWD), has been shown to be a sensitive biomarker for predicting new onset atrial fibrillation and poor prognosis (4). At the same time, the structural parameters provided by cardiac ultrasound are equally crucial: the left atrial volume index (LAVI) serves as a reliable reflection of chronic elevation of left ventricular filling pressure, and its increase indicates the progression of diastolic dysfunction, which is closely related to hospitalization and mortality rates for heart failure (5). In addition, structured cardiac assessment plays a decisive role in revealing potential heart failure (HF) and coronary artery disease in patients with complex conditions such as chronic obstructive pulmonary disease (COPD) (6). These findings collectively point in one direction: based on electrophysiological and anatomical information from routine non-invasive examinations, it is expected to construct a more refined and three-dimensional risk prediction framework (7).

However, current risk stratification tools still have significant limitations. Traditional multivariate prediction models, such as GRACE score or SYS-CAD score, have been widely used in clinical practice, but their core elements are mostly focused on demographic, clinical history, and biochemical indicators, and have not fully utilized the rich information contained in routine ECGs and echocardiograms (8, 9). This has led to a critical information gap: when faced with patients with “abnormal ECG but preserved ejection fraction” or “ultrasound indicating mild left atrial enlargement but normal left ventricular ejection fraction,” clinical doctors often lack precise evidence to quantify their long-term risks (10). The root of this limitation is partly due to the fact that most studies only analyze individual ECG or ultrasound parameters in isolation, ignoring the complex interactions and complementary relationships between the two (11). For example, a meta-analysis clearly indicated that relying solely on normal cardiac imaging results and ignoring abnormal ECG findings may underestimate the true risk of MACE in patients (12). Meanwhile, although the prognostic value of ECG parameters such as QRS duration and Sokolow Lyon voltage (SLV) in specific populations has been validated, there is still a lack of systematic comparative evidence on their independent contribution weights relative to structural parameters (such as LAVI) in multivariate environments (13). For clinically CAD patients, how to organically integrate these discrete indicators and verify their incremental value relative to traditional clinical models is a key bottleneck in optimizing individualized treatment strategies (14, 15).

Therefore, this study aimed to evaluate the independent and combined predictive value of routine ECG parameters (PWD, QRS duration, SLV) and echocardiographic parameters (LAVI, LVMI, LVEF) for long-term MACE in patients with CAD, and to quantify their incremental value over a baseline clinical risk model.

## 2 Data and methods

### 2.1 General information

This study is a single center, observational, retrospective cohort study. The study included patients who were regularly followed up in the cardiovascular department of Jingxian Hospital from January 2024 to February 2026 and had been diagnosed with coronary artery disease (CAD). This research protocol has been approved by the Medical Ethics Committee of the Institutional Review Board of Jingxian Hospital. Due to the retrospective nature of the study and strict protection of patient privacy, the ethics committee waived the requirement to obtain individual informed consent from patients.

### 2.2 Inclusion and exclusion criteria

Inclusion criteria:

1. Age ≥ 18 years old;
2. Meets the above CAD diagnostic criteria: confirmation through invasive coronary angiography (ICA) of at least one major epicardial coronary artery with a lumen diameter stenosis of ≥ 50%, or a clear history of myocardial infarction confirmed by electrocardiographic evolution and dynamic changes in myocardial enzyme spectrum;
3. At baseline assessment (within the enrollment time window), there is a complete record of 12 lead resting standard ECG and transthoracic echocardiography, and the completion time interval between the two examinations is less than 1 week, and both examinations occur before the occurrence of any endpoint event;
4. Have at least one post-baseline clinical follow-up assessment in 6 months, allowing ascertainment of endpoint status or censoring at the data extraction date.

Exclusion criteria:

1. The baseline ECG shows atrial fibrillation, atrial flutter, or ventricular pacing rhythm, which can seriously affect the accurate measurement and interpretation of key ECG parameters such as P-wave dispersion (PWD) and QRS duration;
2. Concomitant severe valvular heart disease, obstructive hypertrophic cardiomyopathy, acute myocarditis, massive pericardial effusion, or known infiltrative cardiomyopathy (such as cardiac amyloidosis, sarcoidosis, etc.) that require surgical intervention, as these diseases themselves significantly alter cardiac electrophysiology and structure, posing a strong competitive risk;
3. End stage renal disease, defined as estimated glomerular filtration rate (eGFR) consistently below 15 mL/min/1.73 m^2^ or undergoing maintenance dialysis treatment due to its extreme impact on electrolytes, cardiac load, and prognosis;
4. The dynamic image quality of echocardiography stored at baseline is poor and cannot meet the requirements of the American Society of Echocardiography (ASE) guidelines for accurate and reproducible measurement of key parameters such as left ventricular ejection fraction (LVEF) and left atrial volume;
5. Missing follow-up or critical follow-up information makes it impossible to accurately determine the endpoint event.

### 2.3 Equipment information

All examinations were conducted using medical equipment that has been regularly quality controlled and calibrated according to the clinical routine configuration of Jingxian Hospital. The resting 12 lead ECG is collected using a MEDEX MECG-200 electrocardiograph, with recording parameters set to standard paper speed of 25 mm/s and gain of 10 mm/mV, operated by experienced physicians in the ECG room. Transthoracic echocardiography is performed using a Philips EPIQ 5 color Doppler ultrasound diagnostic system, equipped with a phased array probe (frequency range 1–5 MHz). All examinations are performed by licensed ultrasound physicians in accordance with standard operating procedures, and complete dynamic loop images are stored in digital format in the hospital’s Picture Archiving and Communication System (PACS) for subsequent offline analysis.

### 2.4 Research methods

1. Baseline clinical data collection: Two cardiology graduate students who have undergone unified training independently extract data from the hospital’s electronic medical record system. The collected contents include: demographic information (age, gender), traditional cardiovascular risk factors (hypertension, diabetes, dyslipidemia, smoking history), past medical history (myocardial infarction, history of revascularization, stroke), baseline drug use (antiplatelet drugs, statins, beta blockers, etc.), laboratory examination results (blood creatinine, lipid profile, etc.), and coronary angiography results (number of diseased vessels, if feasible, record SYNTAX score). After the collection is completed, cross checking will be conducted, and any inconsistencies will be resolved by consulting the original medical records or arbitration by a third senior researcher.
2. Standardized measurement of ECG parameters: All baseline digital ECG files are exported from the ECG network system (such as MUSE Cardiology Information System). The measurement work is carried out on a dedicated workstation equipped with a high-resolution display screen, using calibrated electronic caliper functions. To minimize measurement errors to the greatest extent possible, we have established a strict measurement protocol: all parameters are taken as the average of three consecutive stable sinus rhythm cycles. The specific measurement parameters and methods are as follows: heart rate is taken from the RR interval; PR interval measured in lead II; The QRS wave duration is measured in the chest lead with the widest QRS wave; The QT interval is measured from the beginning to the end point in chest lead V2 or V3, and corrected to QTc using the Bazett formula; The maximum time limit of P-wave (Pmax) and the minimum time limit of P-wave (Pmin) are searched in the limb leads, PWD = Pmax – Pmin; the total QRS wave voltage in limb leads is the algebraic sum of the absolute values of the R and S waves in leads I, II, and III; the Sokolow Lyon voltage is the sum of the maximum values of the S wave depth in lead V1 and the R wave height in lead V5 or V6. All measurements were performed by a cardiologist who was completely unaware of the patient’s subsequent clinical outcomes. To evaluate the repeatability of measurements, 20% of ECGs were randomly selected and independently repeated by another physician using the same method to calculate the intra class correlation coefficient (ICC). Inter-observer reproducibility was high: the ICC for P-wave dispersion was 0.88 (95% CI: 0.82–0.93) and for left atrial volume index was 0.91 (95% CI: 0.86–0.95).
3. Core Laboratory Analysis of Echocardiographic Parameters: According to the latest ASE guidelines, all stored dynamic echocardiographic images were analyzed offline using a senior physician in the cardiac ultrasound core laboratory of Jingxian Hospital who performed blind analysis (on patient clinical information and outcomes). Use quantitative analysis software (such as EchoPAC) that comes with the ultrasonic instrument. The key structural and functional parameters were measured as follows: left ventricular ejection fraction was measured using the biplane Simpson method on the apical four chamber and two chamber views; Measure the end diastolic diameter of the left ventricle, the anterior posterior diameter of the left atrium, the interventricular septum, and the thickness of the left ventricular posterior wall at the end of diastole on the long axis section of the left ventricle adjacent to the sternum; Using the area length method, measure the left atrial volume at the end of ventricular systole on the apical four chamber and two chamber sections, and divide by the body surface area to obtain the left atrial volume index; Apply ASE recommendation formula to calculate left ventricular mass and standardize it as left ventricular mass index; Measure the peak diastolic blood flow velocity (E peak) at the level of the mitral valve apex using pulsed Doppler on the apical four chamber view. Similarly, 30% of the study images were randomly selected for repeated measurements by another core laboratory physician to evaluate inter observer variability.
4. Follow up and determination of endpoint events: The primary endpoint of the study is major adverse cardiovascular events, which is a composite endpoint that includes: (1) cardiogenic death; (2) Non-lethal myocardial infarction (according to the fourth edition of the globally accepted definition); (3) Readmission due to newly diagnosed or acute decompensated heart failure; (4) Unplanned repeated revascularization (including percutaneous coronary intervention or coronary artery bypass grafting) driven by ischemic symptoms or evidence. The identification of endpoint events is completed through system review of electronic inpatient medical records, outpatient follow-up records, and necessary structured telephone follow-up. To ensure the objectivity and accuracy of endpoint determination, we have established an independent endpoint event determination committee composed of two associate chief cardiovascular physicians who were not involved in baseline data measurement and extraction. They independently reviewed and classified each suspected endpoint event based on pre-established standardized definitions, without mutual knowledge of each other’s judgments and without knowledge of baseline ECG and ultrasound parameters. If the two rulings are inconsistent, they shall be submitted to the third chief physician for final arbitration. The follow-up time is calculated from the later date of baseline ECG and echocardiogram examination until the first occurrence of MACE, non-cardiovascular death, loss to follow-up, or study deadline, whichever occurs first.

### 2.5 Observation indicators

The aim of this study is to evaluate the combined predictive value of a series of easily accessible baseline ECG and echocardiogram parameters for the prognosis of CAD patients. The main and secondary observation indicators are as follows:

1. Primary endpoint incidence: Cumulative incidence of major adverse cardiovascular events.
2. Ventricular repolarization parameter: QT interval corrected for heart rate, reflecting the uniformity of ventricular repolarization, and prolonging it is associated with the risk of malignant arrhythmia.
3. Atrial electrical activity parameter: P-wave dispersion, representing the difference in conduction time in different parts of the atrium, is an electrophysiological marker of susceptibility to atrial fibrillation.
4. The core indicator of left ventricular systolic function: left ventricular ejection fraction, is the most widely used indicator for evaluating left ventricular pumping function.
5. Left atrial structural remodeling index: Left atrial volume index, is a sensitive biomarker for left atrial enlargement and long-term elevation of left ventricular filling pressure, closely related to atrial fibrillation and heart failure events.
6. Left ventricular mass parameter: Left ventricular mass index, used to evaluate the degree of left ventricular hypertrophy, is an independent predictor of cardiovascular events.
7. Ventricular conduction parameters: QRS wave duration, reflecting intraventricular conduction velocity, prolongation often indicates the presence of myocardial scars or fibrosis.
8. Ventricular depolarization voltage parameter: Sokolow Lyon voltage, traditionally used for screening left ventricular hypertrophy, recent studies suggest that its decrease may be related to myocardial fibrosis or infiltrative lesions.
9. Overall QRS wave voltage: Low QRS wave voltage in limb leads (total <0.6 mV), often associated with pericardial disease, myocardial infiltration, or severe myocardial injury.

### 2.6 Statistical methods

All statistical analyses were conducted using R language (version 4.3.1) and SPSS Statistics (version 27.0) software. Continuous variables that follow a normal distribution are represented by mean ± standard deviation, and independent sample *t*-test is used for inter group comparison; Non normally distributed variables are represented by the median (25th–75th percentile) using the Mann Whitney *U* test. Categorical variables are expressed in frequency (percentage), and between group comparisons are performed using chi square test or Fisher’s exact test (when expected count < 5). Kaplan Meier method was used to plot survival curves, and the differences between groups were compared using Log rank test.

The determination of the sample size for the study did not involve prior calculations, but instead used all convenient samples that met the established criteria within the study time window. During a median follow-up period of 15.2 months, a total of 50 cases of Major Adverse Cardiovascular Events (MACE) were recorded. According to the empirical rule in multivariate survival analysis that at least 10 events are required for each predictor variable, 50 MACE events are sufficient to support stable estimation of multivariate Cox proportional hazards models containing up to 5 key predictor variables, ensuring the reliability of statistical analysis. Although a wide panel of candidate variables was screened, the final forward stepwise model included only three predictors (diabetes, PWD, LAVI), fulfilling the recommended minimum of 10 events per variable. Bootstrap internal validation was performed to quantify optimism.

In order to identify the independent predictors of MACE, a univariate Cox proportional hazard regression analysis was conducted for all candidate variables (including clinical variables, ECG and echocardiographic parameters), and variables with *p* < 0.1 and clinically recognized important prognostic factors (such as age, diabetes history, and baseline left ventricular ejection fraction) were included in the initial multivariate model. Subsequently, the final multivariate Cox proportional hazards model was constructed using forward stepwise regression (with significance levels of 0.05 for both inclusion and exclusion), and the results were expressed as hazard ratio (HR) and its 95% confidence interval (CI).

In order to quantify the incremental predictive value of ECG and echocardiography parameters on the basis of traditional clinical models, we constructed four nested predictive models: Model 1 (clinical basic model): including age, gender, history of diabetes, previous history of myocardial infarction and estimation of glomerular filtration rate; Model 2: Add significant ECG parameters from univariate analysis to Model 1; Model 3: Add significant echocardiographic parameters from univariate analysis to Model 1; Model 4 (full model): Integrate Model 1 with all significant imaging and electrophysiological parameters. Evaluate the discriminative ability by comparing the Harrell’s C statistic (consistency index) of each model, and use Delong test to determine the statistical significance of the differences in the C statistic. Further calculate the Net Reclassification Improvement (NRI) and Integrated Discrimination Improvement (IDI) indices to evaluate the improvement in predictive performance brought about by the addition of new parameters. The Bootstrap method (1,000 repeated samples) was used for internal validation of the final full model, and the optimistic corrected C statistic was calculated to evaluate the degree of overfitting of the model. Considering that non cardiovascular mortality as a competitive risk may have an impact on the cumulative incidence estimation of MACE, we will use the Fine Gray competitive risk model for sensitivity analysis to validate the robustness of the main Cox analysis results. All hypothesis tests were two-sided, with a *p* value < 0.05 considered statistically significant.

## 3 Results

### 3.1 Baseline characteristics of patients

**Figure 1:**
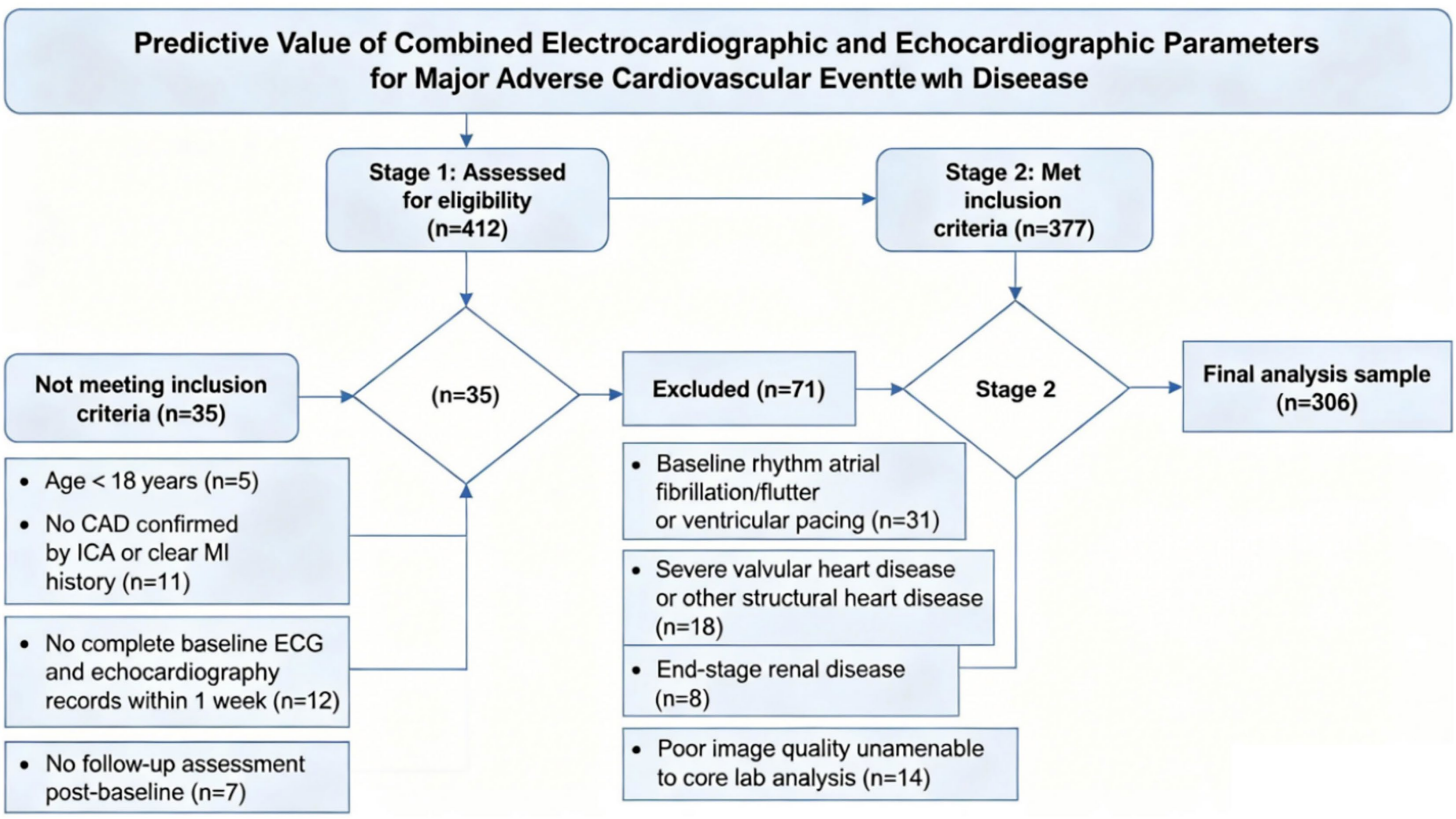
Patient screening and inclusion flowchart.

Through preliminary screening of the hospital’s electronic medical record system, a total of 412 patients who met the initial criteria were recorded. After strict inclusion and exclusion criteria screening (as shown in Figure 1), 306 patients were ultimately included in the final analysis. This study ultimately included 306 patients diagnosed with coronary artery disease (CAD). The median follow-up time was 15.2 months (interquartile range: 9.8–20.1 months), during which a total of 50 major adverse cardiovascular events (MACEs) occurred (cumulative incidence rate of 16.3%). Patients were divided into MACE group (*n* = 50) and non-MACE group (*n* = 256) based on whether MACE occurred. The baseline characteristics comparison between the two groups of patients is shown in Table 1.

**Table 1:** Comparison of baseline characteristics between patients with and without MACE.

Variable	MACE group (*n* = 50)	Non-MACE group (*n* = 256)	*p* value
Demographics			
Age (years), Mean ± SD	64.04 ± 12.79	59.80 ± 10.50	0.012
Male, *n* (%)	36 (72.0)	183 (71.5)	0.941
Cardiovascular risk factors			
Hypertension, *n* (%)	39 (78.0)	179 (69.9)	0.248
Diabetes, *n* (%)	27 (54.0)	85 (33.2)	0.005
Current smoking, *n* (%)	17 (34.0)	72 (28.1)	0.403
Dyslipidemia, *n* (%)	43 (86.0)	204 (79.7)	0.301
Past medical history			
Prior myocardial infarction, *n* (%)	31 (62.0)	103 (40.2)	0.005
Prior revascularization, *n* (%)	33 (66.0)	154 (60.2)	0.438
Laboratory measurements			
eGFR (mL/min/1.73 m^2^), Mean ± SD	71.84 ± 19.02	79.92 ± 17.65	0.004
LDL-C (mmol/L), Median (IQR)	2.24 (1.80, 2.75)	2.12 (1.75, 2.65)	0.41
Coronary lesion characteristics			0.013
Single-vessel disease, *n* (%)	10 (20.0)	92 (35.9)	
Two-vessel disease, *n* (%)	18 (36.0)	100 (39.1)	
Three-vessel disease, *n* (%)	22 (44.0)	64 (25.0)	
SYNTAX score, Median (IQR)	24.5 (17.0, 31.3)	17.0 (11.0, 23.0)	<0.001
Baseline medications			
Aspirin, *n* (%)	48 (96.0)	237 (92.6)	0.381
Statin, *n* (%)	47 (94.0)	232 (90.6)	0.441
Beta-blocker, *n* (%)	41 (82.0)	193 (75.4)	0.313
Electrocardiographic parameters			
QTc (ms), Mean ± SD	445.32 ± 30.05	430.48 ± 27.11	<0.001
PWD (ms), Mean ± SD	45.18 ± 12.07	37.12 ± 10.58	<0.001
QRS duration (ms), Mean ± SD	102.56 ± 16.34	94.89 ± 14.12	<0.001
SLV (mV), Mean ± SD	2.61 ± 0.79	3.05 ± 0.86	<0.001
Limb-lead low voltage, *n* (%)	12 (24.0)	29 (11.3)	0.016
Echocardiographic parameters			
LVEF (%), Mean ± SD	49.82 ± 10.45	56.48 ± 9.12	<0.001
LAVI (mL/m^2^), Mean ± SD	38.77 ± 9.24	32.45 ± 8.45	<0.001
LVMI (g/m^2^), Mean ± SD	118.45 ± 28.34	102.56 ± 24.12	<0.001

Data are presented as mean ± SD, median (IQR), or *n* (%).

Patients in the MACE group were older, and the proportion of diabetes and previous myocardial infarction was significantly higher (*p* < 0.05). In terms of laboratory tests, patients in the MACE group had lower levels of estimated glomerular filtration rate (eGFR). In terms of coronary artery disease, the proportion of three vessel lesions and median SYNTAX score in the MACE group were significantly higher than those in the non-MACE group. There was no significant difference in baseline medication between the two groups. Key electrophysiological and imaging parameters showed that patients in the MACE group had significantly higher corrected QT interval (QTc), P-wave dispersion (PWD), left atrial volume index (LAVI), left ventricular mass index (LVMI), and QRS duration than those in the non-MACE group, while left ventricular ejection fraction (LVEF), Sokolow Lyon voltage (SLV), and total QRS wave voltage in limb leads were significantly lower (all *p* < 0.05).

### 3.2 Primary endpoints and univariate predictive analysis

**Figure 2:**
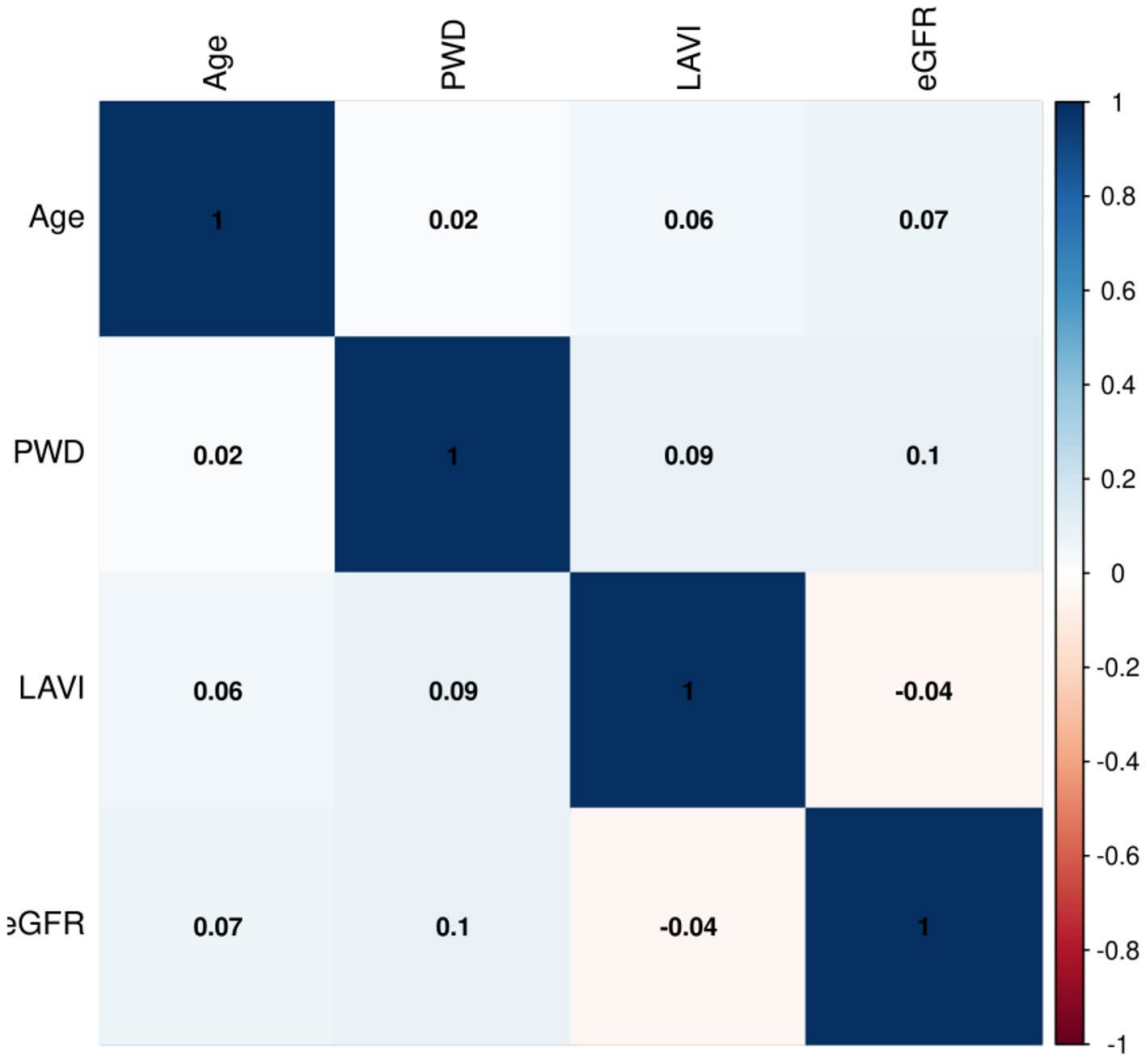
Spearman correlation heatmap of selected electrocardiographic and echocardiographic parameters.

Among the 50 first MACE events, the breakdown was: cardiac death, 8 (16%); non-fatal myocardial infarction, 12 (24%); heart failure readmission, 14 (28%); unplanned repeat revascularization, 16 (32%). Kaplan Meier survival analysis showed that patients with PWD ≥ 40 ms, LAVI ≥ 34 mL/m^2^, and LVEF < 50% had a significantly higher cumulative incidence of MACE (log rank test *p* < 0.001 for all). Univariate Cox proportional hazard regression analysis results (Table 2) showed that age, diabetes history, previous myocardial infarction history, lower eGFR, higher SYNTAX score, QTc prolongation, PWD increase, QRS duration prolongation, SLV decrease, limb lead low voltage, LVEF decrease, LAVI increase, and LVMI increase were significantly related to MACE risk (*p* < 0.1) (Figure 2).

**Table 2:** Univariate and multivariate Cox regression analysis for MACE.

Variable	Univariate HR (95% CI)	Univariate *p* value	Multivariate HR (95% CI)	Multivariate *p* value
Clinical variables				
Age (per 10-year increase)	1.52 (1.18–1.96)	0.001	–	–
Male sex	1.18 (0.66–2.11)	0.556	–	–
Diabetes	2.01 (1.19–3.40)	0.007	1.85 (1.07–3.20)	0.028
Prior myocardial infarction	2.15 (1.27–3.64)	0.003	–	–
eGFR (per 10 mL/min/1.73 m^2^ decrease)	1.28 (1.09–1.50)	0.003	–	–
SYNTAX score (per 5-point increase)	1.22 (1.10–1.35)	<0.001	–	–
Electrocardiographic parameters				
QTc (per 10 ms increase)	1.15 (1.05–1.26)	0.003	–	–
P-wave dispersion (per 10 ms increase)	1.78 (1.42–2.23)	<0.001	1.62 (1.25–2.10)	<0.001
QRS duration (per 10 ms increase)	1.32 (1.12–1.56)	<0.001	–	–
Sokolow-Lyon voltage (per 0.5 mV decrease)	1.45 (1.19–1.77)	<0.001	–	–
Limb-lead low voltage (yes vs. no)	2.12 (1.12–4.01)	0.019	–	–
Echocardiographic parameters				
LVEF (per 5% decrease)	1.31 (1.16–1.48)	<0.001	–	–
LAVI (per 5 mL/m^2^ increase)	1.42 (1.23–1.64)	<0.001	1.30 (1.11–1.52)	0.001
LVMI (per 10 g/m^2^ increase)	1.18 (1.07–1.30)	0.001	–	–

### 3.3 Multivariate Cox regression and joint prediction model

The variables with *p* < 0.1 in univariate analysis and clinically recognized important variables (age, diabetes, LVEF) were included in the multivariate Cox proportional risk model for forward stepwise regression. The final model (Table 2) shows that PWD (HR: 1.62, 95% CI: 1.25–2.10), LAVI (HR: 1.30, 95% CI: 1.11–1.52) and diabetes history (HR: 1.85, 95% CI: 1.07–3.20) are independent predictors of MACE. It is worth noting that although significant in univariate analysis, LVEF and SLV did not maintain independent predictive value in multivariate models.

**Figure 3:**
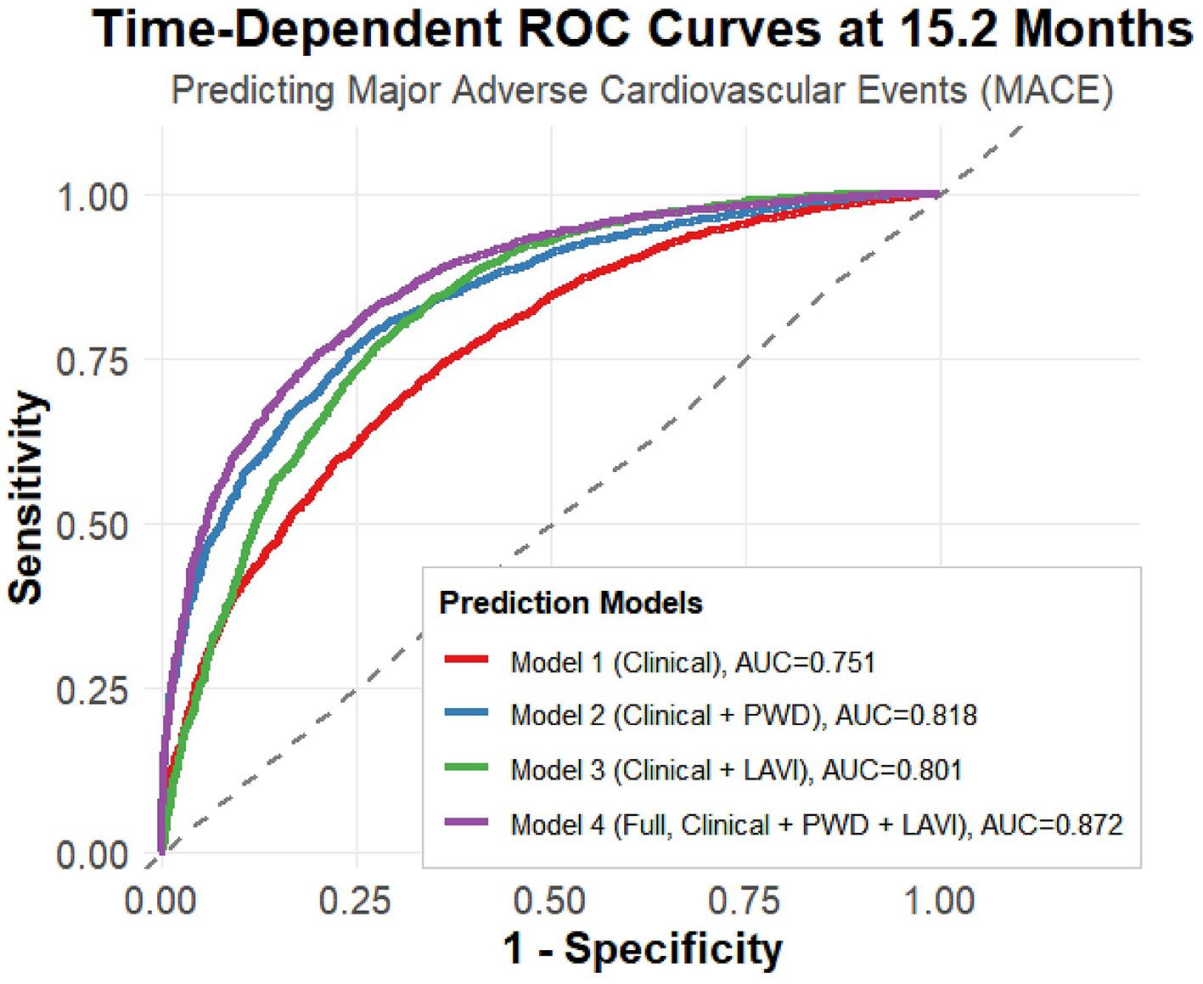
Time-dependent receiver operating characteristic curves for prediction models at 15.2 months.

To evaluate the predictive incremental value of the joint model, we constructed a nested model (Table 3). Compared with the basic model (model 1, C-index = 0.712) that only included clinical variables (age, gender, diabetes, previous myocardial infarction history, eGFR), the full model with PWD and LAVI (model 4) significantly improved predictive discrimination (C-index = 0.791, 95% CI: 0.732–0.850; Delong test, *p* < 0.001). This improvement was further supported by the time-dependent ROC analysis at the median follow-up time of 15.2 months (AUC for Model 4: 0.872, 95% CI: 0.815–0.928). See Figure 3.

**Table 3:** Discrimination and incremental value of different prediction models.

Model	Variables included	C-index (95% CI)	ΔC-index (vs. Model 1)	NRI (95% CI)	IDI (95% CI)
Model 1 (Clinical)	Age, sex, diabetes, prior MI, eGFR	0.712 (0.645, 0.779)	–	–	–
Model 2 (Clinical + ECG)	Model 1 + PWD	0.758 (0.695, 0.821)	0.046	0.312 (0.112, 0.512)^*^	0.045 (0.018, 0.072)^*^
Model 3 (Clinical + Echo)	Model 1 + LAVI	0.745 (0.680, 0.810)	0.033	0.258 (0.085, 0.431)^*^	0.032 (0.010, 0.054)^*^
Model 4 (Full model)	Model 1 + PWD + LAVI	0.791 (0.732, 0.850)	0.079^*^	0.401 (0.205, 0.597)^*^	0.068 (0.035, 0.101)^*^

^*^p < 0.05; the proportional hazards assumption was met for all covariates (Schoenfeld individual test *p* > 0.05). The optimism-corrected C-index for Model 4 was 0.775 (95% CI: 0.710–0.840). Calibration was satisfactory (Grønnesby–Borgan *χ*^2^ = 4.2, *p* = 0.62).

Time-dependent ROC curves for predicting MACE at the median follow-up time of 15.2 months. The AUC values at this specific time point were: Model 1 (Clinical model) = 0.751; Model 2 (Clinical + PWD) = 0.818; Model 3 (Clinical + LAVI) = 0.801; Model 4 (Full model, Clinical + PWD + LAVI) = 0.872. All AUCs were computed within the survival analysis framework. The relative ranking of model performance by time-dependent AUC is concordant with that of the Harrell’s C-index reported in Table 3. Of note, the absolute AUC values are higher than the C-indices, which is expected because the C-index evaluates overall prognostic discrimination across the entire follow-up period, while the time-dependent AUC captures discrimination at a single landmark time point.

### 3.4 Subgroup analysis

In the preset subgroups (gender, age < 65 years vs. ≥ 65 years, presence or absence of diabetes, presence or absence of previous myocardial infarction), the predictive effect of PWD and LAVI in the multivariate Cox model is basically the same. The interaction test showed that there was no significant difference in the correlation between PWD and LAVI and MACE risk among all subgroups (interaction *p*-values were all >0.05), indicating that the effects of these predictive factors are universal in patients with different clinical characteristics. Although no statistically significant interactions were detected, subgroup analyses were performed as exploratory assessments to examine the consistency of effect estimates across clinically relevant strata, supporting the generalizability of the findings.

### 3.5 Sensitivity analysis

To address the potential bias of non-cardiovascular mortality as a competitive risk, we conducted sensitivity analysis using the Fine Gray competitive risk model. The results (Table 4) showed that after adjusting for the competitive risk of non-cardiovascular mortality, PWD (Sub distribution Hazard Ratio, sHR: 1.58, 95% CI: 1.22–2.05) and LAVI (sHR: 1.28, 95% CI: 1.09–1.50) remained independent predictors of MACE, highly consistent with the results of the Cox model, demonstrating the robustness of the research conclusions.

**Table 4:** Subdistribution hazard ratios from fine-gray competing risk regression model.

Variable	sHR (95% CI)	*p* value
Diabetes (yes vs. no)	1.80 (1.04, 3.12)	0.035
PWD (per 10 ms increase)	1.58 (1.22, 2.05)	<0.001
LAVI (per 5 mL/m^2^ increase)	1.28 (1.09, 1.50)	0.003

### 3.6 Adverse events during follow-up period

During the entire follow-up period, a total of 87 adverse events were recorded. Of these, 50 events (16.3%) met the criteria for serious adverse events (SAEs), i.e., resulting in hospitalization, life-threatening conditions, or death. All SAEs were consistent with the primary endpoint (MACE) and included cardiogenic death (*n* = 8), non-fatal myocardial infarction (*n* = 12), rehospitalization for heart failure (*n* = 14), and unplanned repeat revascularization (*n* = 16). The remaining 37 adverse events (12.1%) comprised angina pectoris requiring medication adjustment (*n* = 20), worsening heart failure symptoms not requiring hospitalization (*n* = 8), and new-onset atrial fibrillation (*n* = 9). All SAEs were adjudicated as definitely related to the patient’s underlying cardiovascular disease progression. No adverse events related to the baseline assessments (ECG or echocardiogram) occurred. Detailed information is shown in Table 5.

**Table 5:** Summary of adverse events during follow up period (*n* = 306).

Adverse event category	Events (*n*)	Incidence (%)	Maximum CTCAE v5.0 grade (*n*)	Relationship to disease
Serious adverse events (SAEs)	50	16.3		Definitely related (50)
Cardiac death	8	2.6	Grade 5	Definitely related
Non-fatal myocardial infarction	12	3.9	Grade 4	Definitely related
Rehospitalization for heart failure	14	4.6	Grade 4	Definitely related
Unplanned repeat revascularization	16	5.2	Grade 3	Definitely related
Other adverse events	37	12.1		Probably related (37)
Angina pectoris (requiring medication adjustment)	20	6.5	Grade 2	Probably related
Worsening heart failure symptoms (not requiring hospitalization)	8	2.6	Grade 2	Probably related
New-onset atrial fibrillation	9	2.9	Grade 3	Probably related

## 4 Discussion

One of the core challenges in the long-term management of patients with coronary artery disease (CAD) is to accurately identify subgroups with increased risk of adverse events. Traditional risk stratification tools, such as clinical scoring and single imaging indicators, have certain value, but their predictive accuracy for individualized prognosis is often insufficient (16). This study focuses on the combined predictive efficacy of routine ECG and echocardiography parameters for major adverse cardiovascular events (MACE) (17). From the data point of view, after adjusting for age, diabetes and other traditional risk factors, P-wave dispersion (PWD) and left atrial volume index (LAVI) were confirmed as independent predictors of MACE (18), and the combined model integrating these two parameters significantly improved the risk discrimination ability and reclassification efficiency (19). These findings suggest that the structural and electrophysiological information provided by electrocardiography and echocardiography are complementary in predicting the prognosis of CAD, and their synergistic application may be able to target high-risk populations earlier (20).

Regarding the impact of PWD on prognosis, this study observed that for every 10 milliseconds increase in PWD, the risk of MACE increases by 62%. Behind this difference may be related to the increased heterogeneity of atrial electrical activity (21). The increase in PWD reflects the differences in conduction velocity in different regions of the atrium, and this electrical instability is a known matrix for the occurrence of atrial fibrillation. New onset atrial fibrillation in CAD patients often indicates deterioration of cardiac function and an increase in ischemic events (22). The evidence in the literature supports the view that abnormal ECG responses are associated with an increased risk of long-term MACE in female patients, even in the absence of clear wall motion abnormalities (23). From a mechanistic perspective, an increased PWD is not only a warning signal for arrhythmia, but it may also indirectly reflect an increase in left ventricular filling pressure and atrial structural remodeling, which cannot be ignored (24). Therefore, as an inexpensive and readily available ECG indicator, PWD should be given more weight in clinical risk stratification (25).

The left atrial volume index (LAVI) is an important indicator for measuring the degree of left atrial remodeling. In this study, for every 5 mL/m^2^ increase in LAVI, the risk of MACE increased by 30%, independent of the left ventricular ejection fraction (LVEF) (26). The elevation of LAVI is usually considered a sign of chronic left ventricular filling pressure elevation, which is a compensatory remodeling of the left atrium to counteract the increased afterload (2, 27). Previous work has revealed that routine cardiac assessment can significantly improve the diagnostic rate of heart failure, including left atrial dilation, in patients hospitalized for acute exacerbation of chronic obstructive pulmonary disease (28). This phenomenon is consistent with the results of this study and points to the core role of left ventricular structural and functional abnormalities in prognostic evaluation (29). In contrast, the traditionally highly anticipated LVEF did not enter the final multivariate model in this study, which may be due to LAVI covering more information about diastolic dysfunction, thus to some extent masking the independent weights of LVEF (30).

It is worth noting that the Corrected QT Interval (QTc) and Sokolow Lyon Voltage (SLV), which are significantly correlated with MACE in univariate analysis, did not maintain independent predictive value in multivariate models. QTc prolongation usually reflects abnormal ventricular repolarization, which can easily form a matrix of malignant ventricular arrhythmias. However, the results of this study suggest that when the atrial electrical structural abnormalities represented by PWD and LAVI are included in the model, the predictive effect of QTc is significantly reduced. Similarly, in this study, the MACE group had lower SLV, which is consistent with the trend observed in some studies where a decrease in SLV (<3.5 mV) predicted an increase in adverse events after transcatheter aortic valve replacement surgery. The reduction of SLV may indicate myocardial fibrosis or invasive lesions, but in multivariate models, its effects may be covered by more powerful structural parameters such as LVMI. The complexity of this correlation still needs to be further clarified through integrated analysis of larger samples.

This study reveals an important clinical insight: conventional ECG and echocardiography not only serve for diagnosis, but are also invaluable tools for assessing long-term risk in CAD patients. Relying solely on rough judgments of “normal ECG” or “normal heart function” may miss subtle and critical high-risk warning signals such as increased PWD or LAVI. This viewpoint has been confirmed in the latest literature: even in patients with typical symptoms and initial examination “normal,” clinical suspicion and further structural evaluation are still indispensable, which is crucial to avoid delaying the diagnosis of complex coronary diseases.

In terms of limitations, as a single center, retrospective study with limited sample size and from a single center, this study has inherent selection bias and residual confounding potential, which poses constraints on external generalizability. Although the duration of follow-up and the number of MACE events are sufficient to support multivariate analysis, they may still not be sufficient to reveal long-term, low-frequency differences. The measurement of parameters such as PWD has not yet been automated and unified. Although repeatability has been verified, there may be variations between different devices or operators, and standardized processes urgently need to be established. In addition, this study did not systematically collect data on inflammation or fibrosis biomarkers, which limits the completeness of risk construction validation at multiple dimensions.

In summary, PWD and LAVI are independent and complementary predictors of MACE in CAD patients, and integrating them into traditional clinical models can significantly improve risk stratification efficacy. As a routine parameter with zero additional cost, they have the potential to be embedded in daily practice and assist clinical decision-making. Prospective, multicenter studies are still needed in the future to clarify the defined thresholds and clinical application pathways in different subtypes of CAD.

## Data Availability

The original contributions presented in the study are included in the article/supplementary material, further inquiries can be directed to the corresponding author.
